# Transitional Care Programs for Patients with High Nursing Activity Scores Reduce Unplanned Readmissions to Intensive Care Units

**DOI:** 10.3390/medicina58111532

**Published:** 2022-10-27

**Authors:** Junpei Haruna, Yoshiki Masuda, Hiroomi Tatsumi

**Affiliations:** Department of Intensive Care Medicine, School of Medicine, Sapporo Medical University, Sapporo 060-8556, Japan

**Keywords:** ICU readmission, nursing activities score, transitional care program

## Abstract

*Background and Objectives:* The main objective of a transitional care program (TCP) is to detect patients with early deterioration following intensive care unit (ICU) discharge in order to reduce unplanned ICU readmissions. Consensus on the effectiveness of TCPs in preventing unscheduled ICU readmissions remains lacking. In this case study assessing the effectiveness of TCP, we focused on the association of unplanned ICU readmission with high nursing activities scores (NASs), which are considered a risk factor for ICU readmission. *Materials and Methods*: This retrospective observational study analyzed the data of patients admitted to a single-center ICU between January 2016 and December 2019, with an NAS of >53 points at ICU discharge. The following data were extracted: patient characteristics, ICU treatment, acute physiology and chronic health evaluation II (APACHE II) score at ICU admission, Charlson comorbidity index (CCI), 28-day mortality rate, and ICU readmission rate. The primary outcome was the association between unplanned ICU readmissions and the use of a TCP. The propensity score (PS) was calculated using the following variables: age, sex, APACHE II score, and CCI. Subsequently, logistic regression analysis was performed using the PS to evaluate the outcomes. *Results*: A total of 143 patients were included in this study, of which 87 (60.8%) participated in a TCP. Respiratory failure was the most common cause of unplanned ICU readmission. The unplanned ICU readmission rate was significantly lower in the TCP group. In the logistic regression model, TCP (odds ratio, 5.15; 95% confidence interval, 1.46–18.2; *p* = 0.01) was independently associated with unplanned ICU readmission. *Conclusions*: TCP intervention with a focus on patients with a high NAS (>53 points) may prevent unplanned ICU readmission.

## 1. Introduction

Unplanned readmission to the intensive care unit (ICU) is a common adverse event among critically ill patients. Unplanned ICU readmissions are associated with several negative outcomes such as increased mortality, prolonged ICU stay, and increased costs [1,2]. Previous studies have reported that the rate of ICU readmission is typically between 4 and 11% [3,4,5]. The reported risk factors for unplanned ICU readmission include age [6], severity of illness at ICU admission [7], history of chronic disease [8], ICU length of stay (LOS) [3], catecholamine use [3], and nursing workload, such as that represented by the nursing activities score (NAS) at ICU discharge [9,10]. However, overall ICU readmission rates have changed little over time despite decades of research on ICU readmissions [11].

There have been several attempts to prevent unplanned ICU readmission using predictive tools, such as the Stability and Workload Index for Transfer (SWIFT) score at ICU discharge [12] or the Critical Care Outreach Service [13,14].

Transitional care programs (TCP) after ICU discharge are widely used to support patient care, with a view to preventing unplanned ICU readmissions [15,16,17]. However, the preventive effect of TCP on ICU readmissions remains controversial [18,19,20,21]. Previous reports targeted all enrolled patients after ICU discharge, rather than only those with risk factors for ICU readmission [13,18,22]. 

NAS, developed to measure the nursing workload in ICU, is an index consisting of 7 categories of 23 nursing activities: basic activities, ventilation, cardiovascular, renal, neurological, metabolic support, and other specific interventions [23]. NAS includes nursing care as well as organ support status, which allows for a comprehensive assessment of patients’ conditions. NAS can also be used per shift or day; the sum of the individual items scored can rate the workload spent by the nursing staff on specific time activities in the ICU [24]. NAS at ICU discharge is associated with mortality [25]. Meanwhile, we have previously demonstrated that NAS at ICU discharge is superior to the SWIFT score, while patients with NAS scores ≥ 53 at ICU discharge were at a high risk for ICU readmission [9]; therefore, NAS is effective in assessing nursing workload in ICUs [26].

When a patient is discharged from the ICU with a high NAS, the nursing workload in the general ward will be higher, and healthcare professionals may not be able to provide adequate observation and care for such critically ill patients when discharged from the ICU to the ward. The delayed or inadequate care of patients in the general ward may lead to unplanned ICU readmissions, prolonged hospital stays, cardiac arrest, and death. 

In this study, we focused on patients with a high NAS as potential targets for the effectiveness of TCPs. This study assesses the association between unplanned ICU readmission rates and TCP use in patients with a high NAS.

## 2. Materials and Methods

### 2.1. Design, Setting, and Inclusion Criteria

This retrospective study was conducted at a university hospital. The study design and protocol were approved by the institutional review board of Sapporo Medical University (authorization number: 322-63). Owing to the observational nature of this study, this information was used on an opt-out basis. We enrolled patients with unplanned ICU admissions who survived and were discharged from the ICU between January 2016 and December 2019. We have previously examined the association between unplanned ICU readmission and NAS at ICU discharge and found that the NAS at ICU discharge was significantly higher in the unplanned ICU readmission group. Furthermore, we performed an ROC analysis of NAS with unplanned ICU readmission as the dependent variable and found that the cutoff value was 53 points. Therefore, based on the results of our previous study, this study included patients with an NAS > 53 at ICU discharge, who were considered to be at a high risk of ICU readmission, as previously reported [9]. Unplanned ICU readmission was defined as readmission to the ICU within 7 days of ICU discharge, as previously described [27,28]. 

### 2.2. Exclusion Criteria

Patients who were <18 years of age, died in the ICU, had a Do-Not-Attempt-Resuscitation (DNAR) order, had an ICU LOS < 48 h, had NASs < 53 at ICU discharge, or were missing electronic health record (EHR) data that were required to analyze the NAS at ICU discharge were excluded from this study.

### 2.3. Outcome Measures

The primary outcome was the association between TCP participation and unplanned ICU readmissions. The secondary outcomes were the associations of delirium at ICU discharge and after-hours ICU discharge with unplanned ICU readmission.

### 2.4. Data Collection

The most recent patient data within 24 h prior to ICU discharge were collected from the EHR of our university hospital. Patient characteristics, such as age, sex, and underlying disease, were collected, and the Charlson comorbidity index (CCI), ventilator days, ICU LOS, mortality at 28 days, acute physiology and chronic health evaluation II (APACHE II) score, and sequential organ failure assessment (SOFA) score at ICU admission were calculated using the data from the EHR. 

NAS is an index composed of seven categories, with weights ranging from 1.2 to 32 (Supplementary Materials Table S1), and calculated using the collected data according to the instruction manual [23]. TCPs were started at our institution in January 2018. To evaluate the effectiveness of TCP on unplanned ICU readmission, enrolled patients with an NAS > 53 at ICU discharge were allocated to the non-TCP group (2016–2017) and the TCP group (2018–2019), according to the timing of TCP initiation. 

### 2.5. Criteria for ICU Discharge

Intensivists, attending physicians, and ICU medical staff share the statuses of patients during rounds and meetings held every morning and discuss whether the patient can tolerate ICU discharge according to the guidelines for ICU admission, discharge, and triage [29]. In summary, the criteria for discharge from our ICU are as follows.

(1) A stable condition, which does not require treatment or monitoring that should be performed in the ICU.
(2) Consensus with the attending physician at a multidisciplinary conference, confirming that the patient is ready for ICU discharge.

### 2.6. Description of TCP

The main objective of TCP is to detect deteriorating patients early after ICU discharge in order to reduce unplanned ICU readmissions after ICU stay and discharge to the ward. TCPs are implemented by intensivists, physicians, and nurses in the ICU. Patients with longer than 48 h of ICU stay were selected for TCP participation. Patients discharged with a DNAR order were excluded. TCP team members visit discharged patients in the general ward on weekdays because of limited human resources. Patients targeted for TCP are to receive at least one planned TCP within 72 h of ICU discharge. Each TCP team member’s visit consisted of four parts, as follows: (1) A discussion with TCP members to review any concerns about the patient, (2) a physical examination of the patient, (3) a review of the patient’s recent investigations (laboratory and radiological examinations), and (4) a review of patient care (respiratory care, nutrition, sleep, and mobilization). Following the planned visit, TCP team members provided recommendations to the attending physicians and nurses in the ward. Recommendations are discussed and entered into the EHR as suggestions. A detailed checklist was completed by the TCP team members at each visit. This checklist allowed for a comprehensive assessment of the patient’s condition. The TCP was terminated when patients presented no organ dysfunction at the visit by TCP team members or when the decision was made not to readmit/resuscitate the patient due to clinical deterioration. When follow-up was assessed to be necessary for longer than 72 h, the TCP was continued, and the patient was visited several times. In cases where concerns about a patient were noted during a visit, TCP team members and the attending physician would discuss readmission to the ICU. TCP was initiated in January 2018 and implemented to all patients meeting the above criteria.

### 2.7. Statistical Analysis

Data were assessed for a Gaussian distribution using the Shapiro–Wilk normality test. Normally distributed data were represented as the mean ± standard deviation; otherwise, we used the median and interquartile range, and frequencies and corresponding percentages (%) of categorical variables were recorded. A comparative analysis of the TCP group and the control group (the non-TCP group) of historical cases, from before starting TCPs at our institution, was performed for patient characteristics and clinical data (APACHE II score; mechanical ventilation duration, ICU LOS; CCI; readmission rates; 28-day mortality, among others). Variables were compared using the chi-square test or Fisher’s exact test for categorical variables, while parametric data were compared using the Mann–Whitney *U* test. The propensity score (PS) approach was used to address the selection bias inherent in a retrospective observational study. The PS was calculated by predicted probabilities for the TCP in logistic regression analysis with adjustment for confounding factors (i.e., sex, APACHE II, CCI, and ICU LOS). Subsequently, logistic regression analysis using the PS as the adjusted variable was performed to determine the risk estimate for the association between unplanned ICU readmissions and TCP as the primary outcome or delirium at ICU discharge and after-hours ICU discharge as the secondary outcomes. Statistical significance was set at *p* < 0.05. Statistical analyses were performed using SPSS Statistics version 27 (IBM Corp., Armonk, NY, USA).

## 3. Results

**Figure 1:**
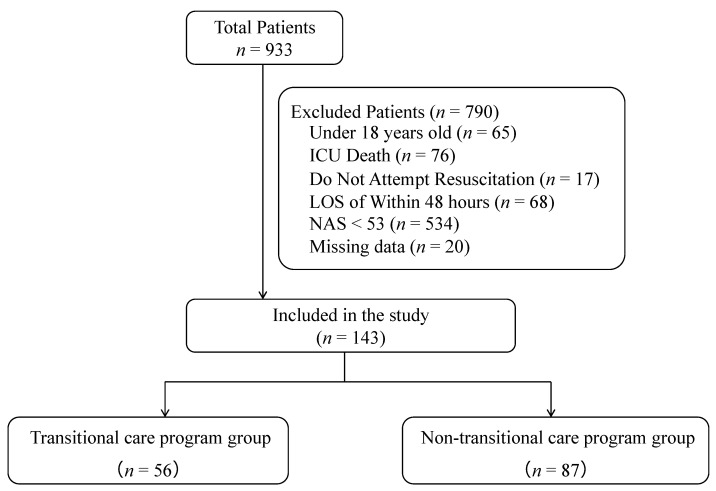
Patient enrollment flowchart.

During the study period, 933 patients were admitted to the ICU, of whom 790 patients were excluded (84.7%); thus, 87 and 56 patients were analyzed in the TCP and non-TCP groups, respectively (Figure 1).

Patient demographics are listed in Table 1. The number of patients unexpectedly readmitted to the ICU was 56 in the TCP group and 87 in the non-TCP group. APACHE II scores were significantly higher in the non-TCP group. Respiratory failure was the most common cause of unplanned ICU readmission in both groups. The average number of TCP team visits was 1.4 times, and the most common consultations were for respiratory support, mobilization, and delirium care, in descending order. The univariate analyses for patients with an NAS < 53 are listed in Supplementary Table S2. There was no significant difference in unplanned ICU readmission in either group.

The results of the logistic regression analysis are presented in Table 2. In the logistic regression model, TCP (odds ratio, 5.15; 95% confidence interval, 1.46–18.2; *p* = 0.01) was independently associated with unplanned ICU readmission. However, after-hours ICU discharge and delirium at ICU discharge were not independent factors for determining unplanned ICU readmission. 

## 4. Discussion

Along with the treatment provided in the ICU, NAS is an objective measure of nursing workload. We have previously found that patients with an NAS > 53 points were at a high risk for unplanned ICU readmission [9]. Subsequently, we conducted the present study to clarify the efficacy of TCPs in preventing unplanned ICU readmission in patients with a high NAS (>53 points). TCP implementation reduced unplanned ICU readmission in patients with a high NAS at ICU discharge. Therefore, implementing TCP after ICU discharge may be more effective for patients with risk factors for unplanned ICU readmission, such as those with a high NAS at ICU discharge.

Previous studies have reported unplanned ICU readmission rates of approximately 10% [30]. Unplanned ICU readmissions remain a significant challenge responsible for increasing mortality and consuming healthcare resources [31]. However, unplanned ICU readmissions do not always reflect medical errors or adverse events and are not always preventable [32]. The early detection and treatment of patients who deteriorate after ICU discharge are recognized as measures for preventing unplanned ICU readmission, and follow-up programs after ICU discharge have been reported [14]. These follow-up programs after discharge from the ICU have been successful in pediatric and adult patients [14,15,17,33]. However, some argue that the effectiveness of follow-up after ICU discharge in adult patients is limited [18,19,20,21,22]. A recent systematic review of TCPs also found limited effectiveness of TCPs in preventing ICU readmission and described the need for interventions targeting patients at high risk of ICU readmission [13]. In the present study, at least one follow-up was performed for patients with a NAS score > 53 points at ICU discharge. Respiratory support was the most common consultation in TCPs, followed by rehabilitation and delirium management. Similar to this study, previous studies have reported respiratory failure as the most frequent reason for unplanned ICU readmission [12,34]. Thus, TCP intervention for continued respiratory support after ICU discharge may be useful in preventing unplanned ICU readmission.

Factors for unplanned ICU readmission from general wards include a lack of detailed care, such as inadequate rehabilitation due to the need to care for inpatients other than those discharged from the ICU, and a failure to recognize patient deterioration at an early stage [7,10]. McQuillan et al. found that approximately 50% of patients with unplanned ICU admissions received substandard care in the general ward, and approximately 41% of those admitted to the ICU may have avoided an ICU admission [35].

Poor quality of care in the general ward not only increases the risk of ICU admission but also increases the risk of rapid deterioration [35,36]. Therefore, there are risks in all aspects of caring for deteriorating patients in general wards. Patients with a high NAS imply increased nursing workloads, which may delay the detection of deterioration. An increased nursing workload has been reported to be associated with higher rates of pneumonia [37], higher mortality rates [38], a higher risk of infection [39], and more medical errors [40]. In addition, care complexity may reduce the time nurses spend collaborating and communicating with physicians, affecting nurse–physician interactions [41]. Moreover, increased nursing workload may decrease nurse–patient communication [42]. In many general wards, resource limitations may hamper the adequate care of patients after discharge from the ICU. If patients with high NASs have to be discharged from the ICU, discharge to a step-down unit, where nurses can provide more adequate care than they can in a general ward, should be considered instead. Furthermore, a TCP can serve as a resource for ward staff to assess patients who are deteriorating and support patients’ recovery after discharge from the ICU. Unplanned ICU readmissions may be prevented when patients are assessed by the TCP team, as they have the necessary skills to recognize the early signs of patient deterioration.

This study had some limitations. First, this study was conducted at a single center; thus, the use of survey results must be carefully implemented in facilities with different characteristics. Second, the study was retrospective, and some of the data were missing from medical records. In addition, TCP interventions were not randomized, which might have caused a selection bias. The strength of this study is that it is one of the few studies conducted in Japan using the NAS at discharge from the ICU to describe unplanned ICU readmission and TCP performance.

## 5. Conclusions

In this study, the influence of TCP implementation on unplanned ICU readmission rates was assessed in patients with an NAS > 53 points at ICU discharge. The results showed that unplanned ICU readmissions were significantly lower when a TCP was implemented. The increased nursing workload for patients at ICU discharge may delay the detection of abnormalities in general wards. TCP intervention with a focus on patients with a high NAS score of >53 points may prevent unplanned ICU readmission. A large-scale prospective study of the efficacy of TCPs in preventing unplanned ICU readmission remains warranted for patients with a high NAS at ICU discharge.

## Figures and Tables

**Table 1:** Patients’ characteristics.

		TCP Group (*n* = 56)	Non-TCP Group (*n* = 87)	*p* Value
Age (years), mean ± SD		67.3 ± 13.1	66.8 ± 12.9	0.88
Male, *n* (%)		32 (57.1)	55 (63.2)	0.49
Charlson Comorbidities Index, mean ± SD		1.6 ± 1.6	1.7 ± 1.4	0.63
APACHE II, mean ± SD		16.5 ± 8.8	20.0 ± 5.9	<0.01
SOFA at ICU admission, mean ± SD		6.1 ± 3.8	6.2 ± 3.1	0.58
Reason for ICU admission				
	Sepsis, *n* (%)	18 (32.1)	28 (32.2)	1.00
	Cardiovascular surgery, *n* (%)	10 (17.9)	11 (12.6)	0.47
	Other surgery, *n* (%)	2 (3.6)	5 (5.7)	0.75
	Respiratory failure, *n* (%)	15 (26.8)	16 (18.4)	0.29
	Circulatory failure, *n* (%)	7 (12.5)	5 (5.7)	0.21
	Cerebrovascular disease, *n* (%)	3 (5.4)	12 (13.8)	0.16
	Acute kidney injury, *n* (%)	2 (3.6)	4 (4.6)	1.00
	Acute pancreatitis, *n* (%)	0 (0)	3 (3.4)	0.28
	Liver failure, *n* (%)	0 (0)	3 (3.4)	0.28
ICU readmission		4 (7.1)	23 (26.4)	<0.01
Reason for ICU readmission				
	Respiratory failure, *n* (%)	3 (75.0)	14 (60.9)	1.00
	Circulatory failure, *n* (%)	1 (25.0)	7 (30.4)	1.00
	Cerebrovascular disease, *n* (%)	0 (0)	1 (4.3)	1.00
	Acute kidney injury, *n* (%)	0 (0)	2 (8.7)	1.00
ICU length of stay (days), mean ± SD		5.8 ± 6.5	7.5 ± 5.0	0.36
Mechanical ventilation, *n* (%)		49 (87.5)	70 (80.5)	0.36
Ventilator days, mean ± SD		5.8 ± 6.5	4.4 ± 4.0	0.69
CRRT, *n*. (%)		16 (28.6)	27 (31.0)	0.85
Mortality for 28 days, *n* (%)		3 (5.4)	5 (5.7)	1.00
Mortality for 90 days, *n* (%)		11 (19.6)	15 (17.2)	0.83
NAS at ICU discharge, mean ± SD		64.9 ± 11.1	66.7 ± 9.8	0.15
Frequency of TCP, mean ± SD		1.4 ± 1.0	-	
Consultation details for TCP				
	Respiratory support, *n*	35	-	
	Mobilization, *n*	18	-	
	Delirium care, *n*	17	-	
	Others, *n*	26	-	

APACHE II, Acute Physiology and Chronic Health Evaluation II; NAS, Nursing Activities Score; CCI, Charlson Comorbidity Index; SOFA, Sequential Organ Failure Assessment; CRRT, Continuous Renal Replacement Therapy; TCP, Transitional care program.

**Table 2:** Odds Ratios for Prediction of Unplanned ICU readmission.

		Odds Ratio (95%CI)	*p* Value
Primary Outcome			
	TCP	5.15 (1.46–18.2)	0.01
Secondary Outcomes			
	Delirium at ICU discharge	0.89 (0.33–2.42)	0.83
	After-hours ICU discharge	0.37 (0.09–1.50)	0.16

TCP, Transitional care program.

## Data Availability

Not applicable.

## Supplementary Materials

The following supporting information can be downloaded at: https://www.mdpi.com/article/10.3390/medicina58111532/s1. Table S1. Nursing Activities Score. Table S2. Patient’s characteristics
