# Supplementary material for: Transitional Care Programs for Patients with High Nursing Activity Scores Reduce Unplanned Readmissions to Intensive Care Units
Source: Medicina (Kaunas). 2022 Oct 27;58(11):1532. doi: 10.3390/medicina58111532 (PMC9693432; doi:10.3390/medicina58111532)
Supplement: Supplementary file 1 [file medicina-58-01532-s001.zip › medicina-1949253-supplementary.pdf]

Supplementary Materials

Transitional Care Programs for Patients with High Nursing Activity Scores Reduce Unplanned Readmissions to Intensive Care Units

Junpei Haruna *, Yoshiki Masuda, and Hiroomi Tatsumi

Department of Intensive Care Medicine, School of Medicine, Sapporo Medical University, Sapporo 060-8556, Japan
* Correspondence: jp.haruna@hotmail.co.jp

Table S1. Nursing Activities Score.

	Basic activities	Score
1	Monitoring and titration	
1a	Hourly vital signs, regular registration, and calculation of fluid balance	4.5
1b	Present at bedside and continuous observation or active for 2 h or more in any shift, for reasons of safety, severity, or therapy such as noninvasive mechanical ventilation, weaning procedures, restlessness, mental disorientation, prone position, donation procedures, preparation and administration of fluids or medication, assisting specific procedures	12.1
1c	Present at bedside and active for 4 h or more in any shift for reasons of safety, severity, or therapy such as those examples above (1b)	19.6
2	Laboratory, biochemical and microbiological investigations	4.3
3	Medication, vasoactive drugs excluded	5.6
4	Hygiene procedures	
4a	Performing hygiene procedures such as dressing of wounds and intravascular catheters, changing linen, washing patient, incontinence, vomiting, burns, leaking wounds, complex surgical dressing with irrigation, and special procedures (e.g., barrier nursing, cross-infection related, room cleaning following infections, and staff hygiene)	4.1
4b	The performance of hygiene procedures took 2 h in any shift	16.5
4c	The performance of hygiene procedures took 4 h in any shift	20
5	Care of drains, all (except gastric tube)	1.8
6	Mobilization and positioning, including procedures such as: turning the patient; mobilization of the patient; moving from bed to chair; team lifting (e.g., immobile patient, traction, prone position)	
6a	Performing procedure(s) up to three times per 24 h	5.5
6b	Performing procedure(s) more frequently than 3 times per 24 h, or with two nurses, any frequency	12.4
6c	Performing procedure with three or more nurses, any frequency	17
7	Support and care of relatives and patient, including procedures such as telephone calls, interviews, counseling; often, the support and care of either relatives or the patient allow staff to continue with other nursing activities (e.g.,	

	communication with patients during hygiene procedures, communication with relatives while present at bedside, and observing the patient)	
7a	Support and care of either relatives or the patient requiring full dedication for approximately 1 h in any shift such as to explain the clinical condition, dealing with pain and distress, difficult family circumstances	4
7b	Support and care of either relatives or the patient requiring full dedication for 3 h or more in any shift such as death, demanding circumstances (e.g., large number of relatives, language problems, and hostile relatives)	32
8	Administrative and managerial tasks	
8a	Performing routine tasks such as processing of clinical data, ordering examinations, professional exchange of information (e.g., ward rounds)	4.2
8b	Performing administrative and managerial tasks requiring full dedication for approximately 2 h in any shift such as research activities, protocols in use, admission, and discharge procedures	23.2
8c	Performing administrative and managerial tasks requiring full dedication for approximately 4 h or more of the time in any shift such as death and organ donation procedures, coordination with other disciplines	30
Ventilatory support		
9	Respiratory support: any form of mechanical ventilation/assisted ventilation with or without positive end-expiratory pressure, with or without muscle relaxants, spontaneous breathing with or without positive end-expiratory pressure with or without endotracheal tube supplementary oxygen by any method	1.4
10	Care of artificial airways: endotracheal tube or tracheostomy cannula	1.8
11	Treatment for improving lung function: thorax physiotherapy, incentive spirometry, inhalation therapy, and intratracheal suctioning	4.4
Cardiovascular support		
12	Vasoactive medication, disregard type, and dose	1.2
13	Intravenous replacement of large fluid losses. Fluid administration 3 $L/m^2/day$, irrespective of type of fluid administered	2.5
14	Left atrium monitoring: pulmonary artery catheter with or without cardiac output measurement	1.7
15	Cardiopulmonary resuscitation after arrest, in the past period of 24 h (single precordial thump not included)	7.1
Renal support		
16	Hemofiltration and dialysis techniques	7.7
17	Quantitative urine output measurement (e.g., by indwelling urinary catheter)	7
Neurologic support		
18	Measurement of intracranial pressure	1.6
Metabolic support		
19	Treatment of complicated metabolic acidosis/alkalosis	1.3
20	Intravenous hyperalimentation	2.8
21	Enteral feeding through gastric tube or other gastrointestinal route (e.g., jejunostomy)	1.3
Specific interventions		
22	Specific intervention(s) in the intensive care unit: endotracheal intubation, insertion of pacemaker, cardioversion, endoscopies, emergency surgery in the previous 24 h, and gastric lavage; routine interventions without direct consequences to the clinical condition of the patient, such as: radiographs, echography, electrocardiogram, dressings, or insertion of venous or arterial catheters, are not included	2.8
23	Specific interventions outside the intensive care unit: surgery or diagnostic procedures	1.9

Table S2. Patient's characteristics

	TCP Group (n = 291)	Non-TCP Group (n = 245)	*P* value
Age (years), mean ± SD	64.8 ± 15.7	61.7 ± 17.9	0.04
Male, *n* (%)	172 (59.1)	137 (55.9)	0.48
Charlson Comorbidities Index, mean ± SD	1.7 ± 1.6	1.8 ± 1.7	0.30
APACHE II, mean ± SD	20.0 ± 5.9	16.5 ± 8.8	< 0.01
SOFA at ICU admission, mean ± SD	6.2 ± 3.1	6.1 ± 3.8	0.96
Reason for ICU admission			
Sepsis, n (%)	52 (17.9)	39 (16.0)	0.64
Cardiovascular surgery, n (%)	44 (15.1)	38 (15.5)	0.91
Other surgery, n (%)	70 (24.1)	61 (24.9)	0.84
Respiratory failure, n (%)	46 (15.8)	42 (17.1)	0.73
Circulatory failure, n (%)	37 (12.7)	34 (13.9)	0.70
Cerebrovascular disease, n (%)	16 (5.5)	18 (7.3)	0.48
Acute kidney injury, n (%)	12 (4.1)	7 (2.9)	0.49
Acute pancreatitis, n (%)	7 (2.4)	10 (4.1)	0.33
Liver failure, n (%)	3 (1.0)	0 (0.0)	0.25
ICU readmission	6 (2.1)	7 (2.9)	0.50
Reason for ICU readmission			
Respiratory failure, n (%)	4 (1.4)	4 (1.6)	1.00
Circulatory failure, n (%)	2 (0.4)	0 (0.00	0.50
Cerebrovascular disease, n (%)	0 (0.0)	1 (0.4)	0.46
Acute kidney injury, n (%)	0 (0.0)	2 (0.8)	0.21
ICU length of stay (days), mean ± SD	3.7 ± 2.5	4.1 ± 2.8	0.07
Mechanical ventilation, n (%)	112 (38.5)	113 (46.1)	0.08
Ventilator days, mean ± SD	1.6 ± 2.2	1.2 ± 2.1	0.04
CRRT, n (%)	34 (11.7)	34 (13.9)	0.52
Mortality for 28 days, n (%)	5 (1.7)	4 (1.6)	1.00
Mortality for 90 days, n (%)	13 (4.5)	13 (5.3)	0.69
NAS at ICU discharge, mean ± SD	38.3 ± 7.2	41.9 ± 6.7	< 0.01
Frequency of TCP, mean ± SD	1.4 ± 0.9	-	
Consultation details for TCP			
Respiratory support, n	125	-	
Mobilization, n	50	-	
Delirium care, n	49	-	
Others, n	84	-	

APACHE Ⅱ, Acute Physiology and Chronic Health Evaluation Ⅱ; NAS, Nursing Activities Score; CCI, Charlson Comorbidity Index; SOFA, Sequential Organ Failure Assessment; CRRT, Continuous Renal Replacement Therapy; TCP, Transitional care program
